# Acute Oral Hydration and Thoracolumbar Fascia Stiffness in Healthy Adults

**DOI:** 10.3390/jcm15145624

**Published:** 2026-07-17

**Authors:** Beata Borecka, Carla Gonçalves, Hsing-Kuo Wang, Dariusz Ciborowski, Tomasz Senderek, Robert Trybulski

**Affiliations:** 1 Physiotherapy Center “Baterie Zdrowia”, Zajączki Drugie, 42-160 Krzepice, Poland; 2 Escola Superior de Desporto e Lazer, Instituto Politécnico de Viana do Castelo, 4900-347 Viana do Castelo, Portugal; carlagoncalves@esdl.ipvc.pt; 3 The Sport Physical Activity and Health Research & Innovation Center, 4900-347 Viana do Castelo, Portugal; 4 School and Graduate Institute of Physical Therapy, National Taiwan University, Taipei 106319, Taiwan; 5 Center of Physical Therapy, National Taiwan University Hospital, Taipei 100229, Taiwan; 6 Body Medica Physiotherapy Center, ul. Górecka 108, 61-483 Poznań, Poland; 7 Department of Rehabilitation and Physiotherapy, Faculty of Health Sciences, Medical University of Lublin, 20-059 Lublin, Poland; 8 Faculty of Medicine, Katowice Business University, 40-659 Katowice, Poland; 9 Provita Medical Centre, 44-240 Żory, Poland

**Keywords:** thoracolumbar fascia, shear wave elastography, hydration, urine specific gravity, bioelectrical impedance analysis

## Abstract

**Objectives**: To determine whether acute oral hydration alters thoracolumbar fascia stiffness in healthy adults and to characterize concurrent changes in urine-specific gravity and bioimpedance-derived fluid compartments. **Methods**: In this prospective single-arm repeated-measures study, 50 healthy adults were assessed at baseline (T0), 60 min after ingestion of 2 L of water (T1), and 6 h later (T2). Thoracolumbar fascia stiffness was measured by shear wave elastography at four standardized points (P1–P4). Hydration was assessed using urine specific gravity and bioimpedance-derived intracellular water, extracellular water, and the extracellular-to-intracellular water ratio. Repeated-measures analyses of variance and Holm-adjusted pairwise comparisons were used. **Results**: Urine specific gravity changed markedly over time (F(2,98) = 290.175, *p* < 0.001, ηp^2^ = 0.856), decreasing from 1.016 ± 0.002 at T0 to 1.006 ± 0.002 at T1, with partial return at T2 (1.014 ± 0.002). Mean thoracolumbar fascia stiffness remained unchanged (45.88 ± 2.01, 45.87 ± 2.01, and 45.89 ± 2.02 kPa at T0, T1, and T2, respectively; F(2,98) = 0.671, *p* = 0.514, ηp^2^ = 0.013). The two-factor shear wave elastography model showed no effect of point (F(3,147) = 2.147, *p* = 0.097) and no time-by-point interaction (F(6,294) = 0.195, *p* = 0.978). Bioimpedance parameters also showed no significant change (all *p* ≥ 0.381). An exploratory correlation between change in urine specific gravity and change in mean stiffness was observed from T0 to T1 (r = −0.346, *p* = 0.028). **Conclusions**: In this single-arm repeated-measures study, acute oral water ingestion was followed by a clear urinary hydration response but by no detectable short-term within-participant change in thoracolumbar fascia stiffness or bioimpedance-derived fluid distribution in healthy adults. Because no non-hydration or placebo control condition was included, these findings should be interpreted as evidence of temporal stability under the tested protocol rather than as a definitive controlled estimate of the causal effect of hydration. Clinical Registration: ISRCTN96046168 (31 March 2026).

## 1. Introduction

The fascial system is a continuous three-dimensional connective-tissue network that contributes to force transmission, postural support, proprioception, and coordinated movement [1,2]. Within this network, the thoracolumbar fascia is especially relevant because it mechanically links trunk and pelvic structures and participates in lumbar load transfer [1]. Interest in the thoracolumbar fascia has increased because altered fascial mobility, shear behavior, and nociceptive innervation have been implicated in musculoskeletal pain, particularly chronic low back pain [2,3,4]. Accordingly, identifying modifiable determinants of thoracolumbar fascial mechanical behavior has become a relevant question in musculoskeletal research and rehabilitation [5,6].

Fascial mechanics depend not only on collagen architecture but also on extracellular-matrix composition, including hyaluronan-rich ground substance that facilitates sliding between adjacent fascial layers [7,8,9]. Fasciacytes and hyaluronan-rich loose connective tissue provide a biologically plausible basis for linking tissue hydration with fascial gliding, but this relationship cannot be inferred directly from mechanistic or histologic evidence alone [8,10,11]. Accordingly, direct in vivo studies are needed to determine whether systemic hydration changes translate into measurable changes in fascial mechanical behavior [6,8]. Shear wave elastography provides a quantitative ultrasound-based approach to assess fascial stiffness in vivo, whereas urine specific gravity and multi-frequency bioelectrical impedance offer practical complementary indicators of systemic hydration and fluid distribution [6,12,13,14].

Recent imaging studies indicate that thoracolumbar fascia stiffness measured by elastography varies with posture and mechanical loading, and may also differ between individuals with and without chronic low back pain [15,16,17]. Methodological work in healthy adults has shown good-to-excellent reproducibility for thoracolumbar fascial shear-modulus measurements, supporting the feasibility of repeated-measures designs [12,18]. Intervention studies likewise suggest that some local mechanical inputs, such as myofascial techniques, can induce immediate changes in lumbar fascial or related soft-tissue parameters, although findings are not uniform across protocols and outcomes [18,19,20]. In parallel, hydration research has established urine specific gravity as a practical biomarker and has shown that bioimpedance-derived estimates can characterize whole-body fluid compartments, but these approaches do not directly quantify local fascial mechanical behavior [13,14,21,22,23].

Consequently, an important knowledge gap persists at the intersection of hydration physiology and fascial biomechanics [6,24]. Most thoracolumbar fascia imaging studies have focused on pain states, posture, or mechanically targeted interventions rather than controlled systemic hydration manipulation [6,15,16,19]. Direct human evidence testing whether an acute water load is accompanied by short-term changes in thoracolumbar fascial stiffness therefore remains limited [6,7,8].

Against this background, the present study was designed to examine whether acute ingestion of 2 L of water alters thoracolumbar fascia stiffness in healthy adults across short-term repeated assessments. The primary objective was to quantify time-dependent changes in shear wave elastography measurements obtained at standardized thoracolumbar sites, and the secondary objectives were to characterize concurrent changes in urine specific gravity and bioimpedance-derived fluid compartments and to explore the relationship between hydration-marker changes and stiffness changes. We hypothesized that acute oral hydration would produce measurable changes in urinary hydration status and might be accompanied by reduced thoracolumbar fascial stiffness over the short-term observation period [7,8,24].

## 2. Materials and Methods

### 2.1. Study Design and Reporting Framework

This prospective, single-arm interventional study used a within-participant repeated-measures design to evaluate the short-term effects of acute hydration on thoracolumbar fascia stiffness and selected hydration markers in healthy adults. Each participant served as his or her own control. This design was selected because thoracolumbar fascia stiffness measurements can vary substantially between individuals, whereas repeated assessment of the same participant reduces between-person variability and increases sensitivity to short-term within-participant physiological change. However, single-arm before–after and repeated-measures designs cannot fully distinguish intervention-related effects from temporal variation, repeated-measurement effects, regression to the mean, or other time-varying influences. Therefore, this design was considered appropriate for detecting within-participant temporal patterns but not for definitive causal attribution [25,26]. Measurements were obtained at baseline (T0), at the protocol-defined early post-hydration assessment (T1), and 6 h after the intervention (T2). A parallel non-hydration or placebo condition was not included because the study was designed as an initial physiological challenge to determine whether a supervised 2 L oral water load produced a measurable hydration response and whether any concurrent short-term change in thoracolumbar fascia stiffness was detectable under standardized resting conditions. Accordingly, all findings are interpreted as within-participant temporal observations following the hydration protocol rather than as controlled estimates of the isolated causal effect of hydration.

### 2.2. Setting, Ethics, and Registration

All procedures were conducted at Provita Medical Center under standardized testing conditions. The protocol was approved by the Ethics Committee of the Scientific Research of Physiotherapists at the Polish Physiotherapy Association (resolution no. 6/03/2026; 20 March 2026). The study was conducted in accordance with the Declaration of Helsinki. All participants provided written informed consent before enrollment. The protocol was prospectively registered in the ISRCTN registry under identifier ISRCTN96046168 (registration date: 31 March 2026).

### 2.3. Participants

A priori sample size estimation was based on the primary analysis, namely the within-subject effect of time on global thoracolumbar fascia stiffness across the three repeated assessments (T0, T1, and T2) [27]. The calculation was performed in G*Power 3.1 under the F tests family using ANOVA: repeated measures, within factors [28]. Because the thoracolumbar fascia ultrasound literature remains relatively sparse and methodologically heterogeneous, and published work has focused mainly on measurement properties and mechanically induced stiffness changes rather than hydration manipulation, an exact hydration-specific effect size was not available from prior human studies [6]. The closest human shear wave elastography measurement of thoracolumbar fascia stiffness (SWE) studies have shown that thoracolumbar fascia stiffness can be measured reproducibly and can change under posture- or load-related conditions, while more recent cross-sectional work has demonstrated measurable stiffness differences between healthy individuals and patients with chronic nonspecific low back pain [12,15,16]. Because those paradigms involve stronger mechanical contrasts than a short-term oral hydration challenge, powering the present study for a large or even clear moderate effect would have risked overestimating the most plausible intervention signal. Therefore, a conservative small-to-moderate standardized effect size of Cohen’s f = 0.20 was prespecified as the minimum effect [29]. For transparency, this value corresponds to a partial eta-squared of approximately 0.038 $\eta p^{2}=f^{2}/(1+f^{2})$, which is appropriately conservative for an exploratory physiological intervention expected to have subtler effects than posture- or contraction-based loading [29]. Assuming a two-sided α = 0.05, power = 0.80, three repeated measurements, an average correlation among repeated measurements of 0.50, and a conservative nonsphericity correction ε = 0.80, the repeated-measures ANOVA calculation yields df1 = (m − 1)ε = 1.6, df2 = (N − 1)(m − 1)ε = 1.6(N − 1), and a noncentrality parameter of λ = Nm f^2^ ε/(1 − ρ) = N × 3 × 0.20^2^ × 0.80/0.50 = 0.192N, resulting in a required analyzable sample of 49 participants.

Healthy adults were recruited by convenience sampling. Inclusion criteria were age 20–45 years, absence of thoracolumbar or lumbar pain during the previous 6 months, no history of spinal surgery, and ability to comply with the study protocol. Exclusion criteria comprised neurological, musculoskeletal, or systemic conditions that could affect tissue mechanical properties or hydration status, as well as use of medications that could alter fluid balance. Fifty participants were enrolled and completed the study, including 28 men and 22 women. Age, body mass, height, and body mass index were recorded at baseline. The analysis population comprised all enrolled participants because complete measurements were available for all prespecified outcomes at T0, T1, and T2.

### 2.4. Pre-Assessment Standardization and Hydration Intervention

Participants were instructed to maintain their usual fluid intake during the 24 h preceding testing and to avoid deliberate overhydration. They were required to abstain from alcohol for at least 24 h and from caffeine-containing beverages for at least 12 h before baseline testing. To standardize baseline status, participants refrained from fluid intake for at least 2 h before T0. All assessments for a given participant were performed at the same time of day. The intervention consisted of supervised ingestion of 2 L of still, non-carbonated water over 60 min. This fixed absolute volume was selected as a standardized acute water-loading challenge intended to create a clear short-term systemic hydration stimulus while keeping the protocol operationally consistent across participants. The protocol was not designed as an individualized rehydration prescription and was not normalized to body mass, sex, baseline hydration status, or estimated total body water; therefore, the same 2 L volume may have represented different relative physiological stimuli across participants [30,31].

Follow-up measurements were obtained at the protocol-defined early post-hydration assessment (T1) and again 6 h after the intervention (T2). Between T1 and T2, participants were asked to avoid additional fluid intake, strenuous physical activity, and behaviors likely to materially alter fluid balance, while otherwise continuing routine daily activities.

### 2.5. Outcome Measures and Blinding

Assessments were conducted in the morning, in a dedicated laboratory with a temperature of 22 °C and relative humidity of 55%. Outcome assessment comprised urine specific gravity, bioelectrical impedance analysis, and shear wave elastography. Separate assessors with expertise in the respective modality performed each measurement domain. Assessors were blinded to the study hypotheses and were not informed whether a given assessment corresponded to baseline or follow-up. Data were coded before statistical analysis, and the analyst was blinded to measurement sequence during the primary statistical workflow.

### 2.6. Urine Specific Gravity

Hydration status was assessed using freshly collected urine samples and a handheld optical clinical refractometer with automatic temperature compensation (REC-200ATC, Gain Express Holdings Ltd., Hong Kong, China; year of manufacture, 2024). The handheld optical refractometer operates on the principle of light refraction and provides direct measurement of urine specific gravity over a range of 1.000–1.050, with a scale division of 0.005. The instrument is calibrated at a reference temperature of 20 °C and incorporates automatic temperature compensation over an operating range of 10–30 °C to account for temperature-related variation in refractive measurements. Previous studies have demonstrated strong agreement and analytical validity of refractometer-derived measurements compared with reference laboratory methods, as well as greater accuracy than urine dipstick testing [13,32,33]. Furthermore, urine specific gravity assessment using refractometry is widely used as a rapid, validated indicator of hydration status in both clinical and field-based research settings [13].

For each assessment, 1–2 drops of urine were placed on the prism and urine specific gravity was read immediately according to the manufacturer’s instructions. Urine specific gravity was analyzed as a continuous dimensionless variable, with lower values indicating more dilute urine.

### 2.7. Shear Wave Elastography (SWE)

**Figure 1:**
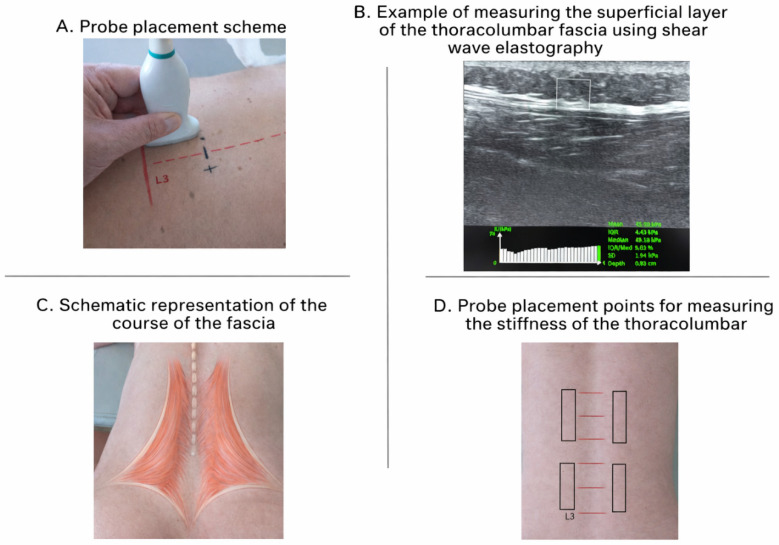
(**A**) probe placement scheme; (**B**) example of region of interest placement within the superficial thoracolumbar fascia during shear wave elastography acquisition; (**C**) schematic representation of the course of the fascia; (**D**) probe placement points for measuring the stiffness of the thoracolumbar fascia. The probe was positioned over standardized anatomical points, and region of interest placement was restricted to the visible fascial layer.

Thoracolumbar fascia stiffness was assessed using shear wave elastography with an ultrasound system (SonoScape P30, SonoScape Medical Corp., Shenzhen, China; year of manufacture 2023) equipped with a SonoScape 3–18 MHz linear-array transducer (Figure 1A). Shear wave elastography is considered a reliable and reproducible method for assessing soft tissue mechanical properties, including fascia. Previous work has demonstrated high intra- and inter-rater reliability for thoracolumbar fascia shear wave elastography when acquisition position and measurement location are standardized [12]. Because SWE estimates can be influenced by technical factors including acquisition depth, transducer pressure, tissue precompression, and region of interest (ROI) placement, the acquisition procedure was standardized before each measurement.

All measurements were performed with participants in a standardized prone position. Participants were instructed to remain relaxed and to avoid voluntary contraction of the trunk and paraspinal muscles during image acquisition. Four sites (P1–P4) were marked approximately 2 cm lateral to the spinous process line between T11 and L3 (Figure 1D). The same participant position, anatomical landmarks, transducer, SWE preset, ROI size, and measurement approach were used at all three time points. A 1 cm × 1 cm region of interest was positioned within the superficial thoracolumbar fascia using the system measurement scale (Figure 1B). The lower boundary of the region of interest was placed tangentially to the interface between subcutaneous tissue and the underlying paraspinal musculature to restrict sampling to the fascia (Figure 1C). Measurement depth was individualized according to subcutaneous tissue thickness and was typically 1.5–2.0 cm.

Image quality was checked first in B-mode to confirm clear visualization of the superficial thoracolumbar fascia, the subcutaneous tissue–fascia interface, and the underlying paraspinal muscle boundary. SWE images were accepted only when the fascia was clearly identifiable, the ROI was fully contained within the intended fascial layer, the elastography map was stable, and no visible motion artifact, signal dropout, or substantial ROI displacement was present [34,35]. All SWE examinations were performed by a single experienced operator using the same ultrasound system, transducer, participant position, anatomical landmarks, SWE preset, and region of interest placement procedure at all three time points. To minimize operator-induced tissue compression, the transducer was applied with the lightest pressure sufficient to maintain acoustic coupling, using ultrasound gel and avoiding visible indentation of the skin, displacement of the subcutaneous layer, or deformation of the fascial plane [34,35]. Using one operator was intended to reduce inter-operator variability in probe handling and transducer pressure. However, transducer pressure was standardized qualitatively rather than quantified with a force sensor or pressure-feedback device.

Stiffness was expressed in kilopascals (kPa). For the global shear wave elastography outcome, the four site-specific measurements were averaged at each time point, while point-specific values were retained for spatially repeated-measures analyses. Standardized positioning, repeated identification of the same anatomical landmarks, consistent placement of the region of interest, predefined image-quality criteria, and blinded analysis of coded data were used to maximize measurement consistency across time points. A formal pilot assessment of within-session intra-rater repeatability was conducted in a subsample of 20 participants and yielded a mean intraclass correlation coefficient of 0.93. Accordingly, measurement precision was evaluated based on the standardized acquisition procedures used in the present study, together with previously published reliability data for shear-wave elastography of the thoracolumbar fascia [12].

### 2.8. Bioelectrical Impedance Analysis

Body fluid distribution was assessed using a multi-frequency bioelectrical impedance analyzer (ACCUNIQ BC720, SELVAS Healthcare Inc., Daejeon, Republic of Korea; year of manufacture 2020) using six measurement frequencies: 1, 5, 50, 250, 550, and 1000 kHz. Bioelectrical impedance analysis is widely recognized as a reliable and reproducible method for assessing body composition and hydration status. Previous studies have demonstrated high test–retest reliability and good agreement of multi-frequency BIA devices in estimating total body water and its compartments [36,37]. Multi-frequency BIA, in particular, has been shown to provide improved accuracy in differentiating intracellular and extracellular fluid compartments compared to single-frequency methods [37].

Measurements were obtained in the standing position according to the manufacturer’s protocol under standardized conditions. Participants were instructed to avoid food intake, vigorous exercise, and excessive fluid intake before measurement. Based on impedance measurements, the device estimates intracellular water (ICW) and extracellular water (ECW), expressed as percentages (%), as well as the extracellular-to-intracellular water ratio (ECW/ICW), expressed as a dimensionless value. Intracellular water, extracellular water, and the extracellular-to-intracellular water ratio were extracted for analysis and treated as continuous variables.

### 2.9. Statistical Procedures

All analyses were conducted to address within-participant changes across the three prespecified assessments: baseline (T0), 60 min after completion of the hydration intervention (T1), and 6 h after the intervention (T2). The analytic sample comprised all enrolled participants with complete repeated-measures data. Because no observations were missing, no imputation procedures were required. Continuous variables were inspected graphically and by standardized values within each time point.

Descriptive statistics are reported as mean ± standard deviation for continuous variables and as counts with percentages for categorical baseline characteristics. Distributional assumptions were evaluated using histograms, quantile inspection, and Shapiro–Wilk tests of within-subject change scores. For the primary outcomes, urine specific gravity and mean shear wave elastography stiffness, change-score distributions were approximately normal. One-factor repeated-measures ANOVA with time as the within-subject factor was used for urine specific gravity, intracellular water, extracellular water, the extracellular-to-intracellular water ratio, and mean thoracolumbar fascia stiffness averaged across the four measurement points. When the omnibus time effect was significant, post hoc pairwise comparisons were performed for T1 versus T0, T2 versus T0, and T2 versus T1 using paired t tests, with multiplicity controlled within outcome by the Holm procedure. Pairwise contrasts were expressed as the later minus the earlier time point, and each contrast is reported with its 95% confidence interval.

Shear wave elastography measurements obtained at P1–P4 were analyzed primarily with a two-factor repeated-measures ANOVA including time (T0, T1, T2) and measurement point (P1–P4) as within-subject factors. This model was used to test for a global temporal effect, a spatial effect across measurement points, and a time-by-point interaction. To assess whether any local temporal effect was masked in the global model, exploratory point-specific repeated-measures ANOVAs were additionally fitted for P1–P4, with Holm adjustment across the four point-specific tests. Because the study had a single-arm within-subject design, no between-group comparison was applicable. Consequently, the repeated-measures analyses quantify temporal change within participants after the hydration protocol, but they do not estimate a controlled treatment effect relative to a non-hydration or placebo condition. Potential time-related measurement effects, repeated positioning or measurement effects, regression to the mean, and other uncontrolled physiological variation therefore could not be statistically separated from the hydration exposure in this design [25].

Effect sizes for omnibus repeated-measures ANOVAs are reported as partial eta squared (ηp^2^). Pairwise within-subject effects are reported as Cohen’s dz, with the direction of change conveyed by the corresponding mean difference. Exploratory associations between change in hydration and change in fascial stiffness were assessed using Pearson correlations between the change in urine specific gravity and the concurrent change in mean thoracolumbar fascia stiffness from T0 to T1 and from T0 to T2; 95% confidence intervals for the correlation coefficients were derived using Fisher’s z transformation, and *p* values were Holm adjusted across the two exploratory correlations. All tests were two-sided with a significance threshold of α = 0.05. Exact *p* values are reported when *p* ≥ 0.001, and smaller values are reported as *p* < 0.001. All analyses were performed in Python 3.13.5 using pandas 2.2.3, SciPy 1.17.0, statsmodels 0.14.6, and Matplotlib 3.10.8.

## 3. Results

**Figure 2:**
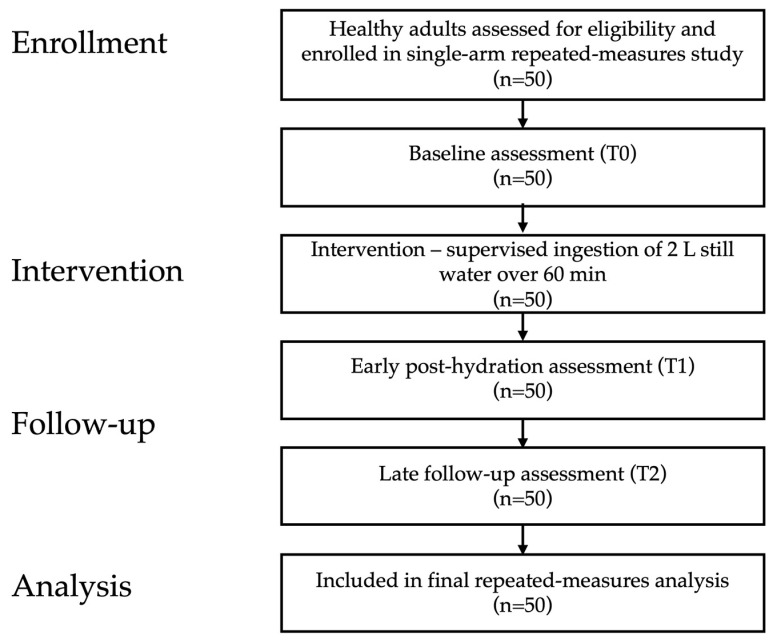
Flow chart. All participants received the hydration protocol. No parallel non-hydration or placebo comparator group was included.

All 50 enrolled participants completed the T0, T1, and T2 assessments and were included in the final analysis; thus, there was no attrition and no missing outcome data (Figure 2). The analyzed sample comprised 28 males and 22 females, with a mean age of 34.3 ± 7.6 years and a mean body mass index of 29.0 ± 5.9 kg/m^2^ (Table 1). An exploratory sex-stratified sensitivity analysis based on change scores showed no evidence that sex modified the temporal response in urine specific gravity, intracellular water, or extracellular water (all *p* ≥ 0.50). Two nominal between-sex differences were observed for the change in mean SWE from T0 observed for the change in mean SWE from T0 to T1 and for the change in ECW/ICW from T0 to T2. However, these did not remain significant after Holm correction for multiple exploratory comparisons.

In contrast, the bioimpedance measures did not show evidence of time-dependent change. Intracellular water remained essentially unchanged (30.99 ± 0.69% at T0, 30.98 ± 0.70% at T1, and 31.00 ± 0.69% at T2; F(2,98) = 0.814, *p* = 0.446, ηp^2^ = 0.016). The same pattern was observed for extracellular water (18.01 ± 0.28%, 18.01 ± 0.31%, and 18.00 ± 0.28%; F(2,98) = 0.974, *p* = 0.381, ηp^2^ = 0.019) and for the extracellular-to-intracellular water ratio (0.361 ± 0.001, 0.361 ± 0.002, and 0.361 ± 0.002; F(2,98) = 0.103, *p* = 0.902, ηp^2^ = 0.002) (Table 2). All pairwise bioimpedance changes were small in magnitude and remained non-significant after Holm correction (all adjusted *p* ≥ 0.586; all dz ≤ 0.186).

**Figure 3:**
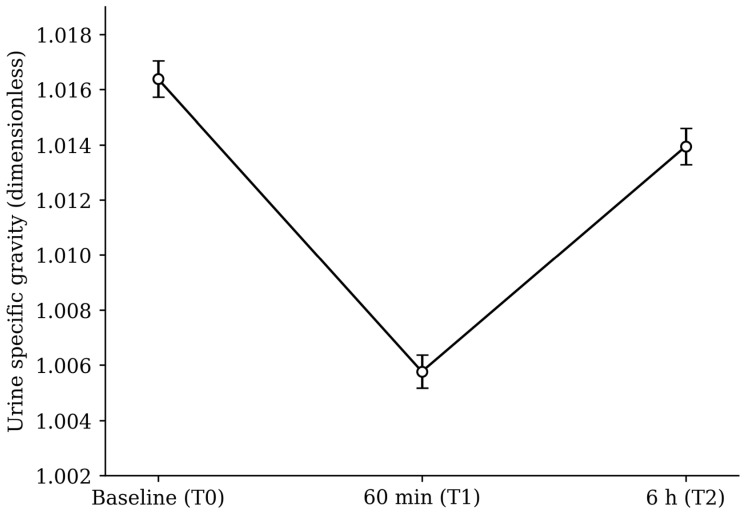
Urine specific gravity across the three study time points. Points represent means and error bars indicate 95% confidence intervals. Lower values indicate more dilute urine and therefore a stronger hydration response.

Urine specific gravity changed significantly over time (F(2,98) = 290.175, *p* < 0.001, ηp^2^ = 0.856), decreasing from 1.016 ± 0.002 at T0 to 1.006 ± 0.002 at T1, with partial return toward baseline by T2 (1.014 ± 0.002) (Table 2; Figure 3). Pairwise comparisons confirmed a large decrease from T0 to T1 (mean change T1 − T0 = −0.0106, 95% CI −0.0115 to −0.0097, Holm-adjusted *p* < 0.001, dz = 3.394), a smaller but still significant reduction at T2 relative to T0 (−0.0024, 95% CI −0.0035 to −0.0014, Holm-adjusted *p* < 0.001, dz = 0.673), and recovery between T1 and T2 (+0.0082, 95% CI 0.0073 to 0.0090, Holm-adjusted *p* < 0.001, dz = 2.719) (Table 3).

**Figure 4:**
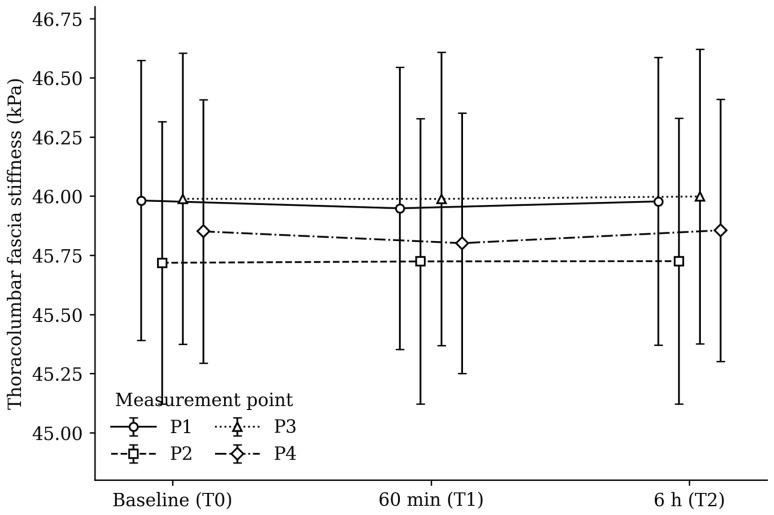
Thoracolumbar fascia stiffness at each measurement point across time. P1–P4 denote the four standardized shear wave elastography locations. Points represent means and error bars indicate 95% confidence intervals.

Mean thoracolumbar fascia stiffness averaged across P1–P4 was 45.88 ± 2.01 kPa at T0, 45.86 ± 2.01 kPa at T1, and 45.89 ± 2.02 kPa at T2, with no evidence of change over time (F(2,98) = 0.671, *p* = 0.514, ηp^2^ = 0.013) (Table 2). Consistent with the primary two-factor shear wave elastography model, there was no significant main effect of measurement point (F(3,147) = 2.147, *p* = 0.097, ηp^2^ = 0.042) and no time-by-point interaction (F(6,294) = 0.195, *p* = 0.978, ηp^2^ = 0.004), indicating absence of both global and site-specific stiffness modification after acute hydration. Point-specific exploratory analyses corroborated this finding: none of the four local measurements showed a time effect after multiplicity correction (all Holm-adjusted *p* > 0.999; all ηp^2^ ≤ 0.015) (Table 4; Figure 4). Pairwise contrasts for mean thoracolumbar fascia stiffness were likewise negligible, with mean changes ranging from −0.02 to 0.02 kPa and trivial standardized effects (all adjusted *p* > 0.999; all dz ≤ 0.130) (Table 3).

**Figure 5:**
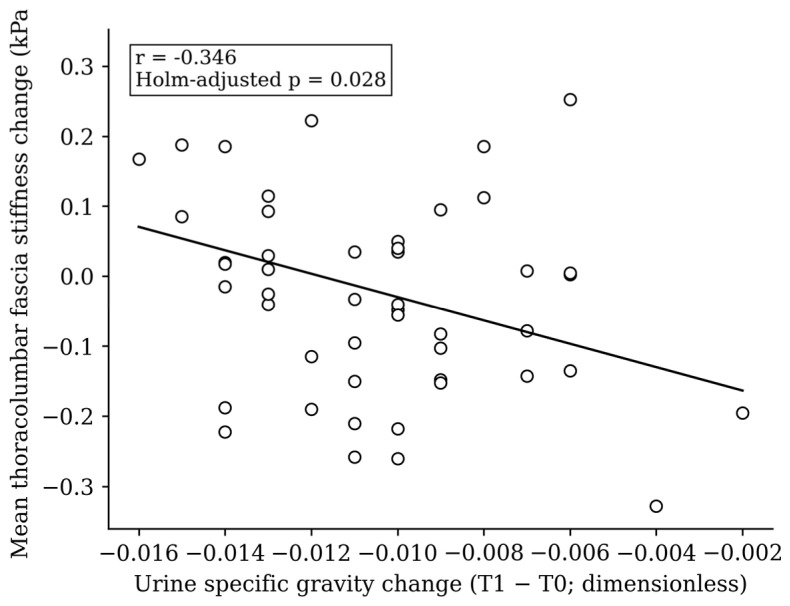
Association between the change in urine specific gravity and the concurrent change in mean thoracolumbar fascia stiffness from baseline to 60 min. Each circle represents one participant; the solid line is the least-squares regression line. More negative values on the x-axis indicate a larger reduction in urine specific gravity.

In exploratory correlation analyses, the change in urine specific gravity from T0 to T1 showed a modest association with the concurrent change in mean thoracolumbar fascia stiffness (r = −0.346, 95% CI −0.569 to −0.075, Holm-adjusted *p* = 0.028) (Figure 5). Because more negative values for urine specific gravity change denote greater urine dilution, this coefficient indicates that participants with larger acute reductions in urine specific gravity tended to show slightly higher or less negative concurrent changes in mean thoracolumbar fascia stiffness. No corresponding association was present for the change from T0 to T2 (r = 0.027, 95% CI −0.254 to 0.303, Holm-adjusted *p* = 0.854).

## 4. Discussion

The present study showed that acute ingestion of 2 L of water produced a meaningful change in urine specific gravity but did not alter thoracolumbar fascia stiffness, whether stiffness was analyzed as a global mean or at any of the four standardized measurement points. Bioimpedance-derived indices of intracellular and extracellular water likewise remained stable, whereas the exploratory correlation between early urine dilution and early stiffness change was modest and not reproduced at the later assessment. These findings indicate that the protocol was accompanied by a clear urinary hydration response but, because no non-hydration or placebo condition was included, they should be interpreted as showing no detectable within-participant stiffness change across the observed post-ingestion period rather than as definitive evidence that hydration cannot causally influence thoracolumbar fascial mechanics.

A relevant physiological distinction is that the three measurement domains used in this study reflect different biological compartments. Urine specific gravity primarily reflects renal urine concentration and systemic water-handling rather than direct hydration of the thoracolumbar fascial extracellular matrix [38]. Bioelectrical impedance analysis provides model-derived estimates of whole-body or segmental fluid compartments, but it does not directly quantify water content within the superficial thoracolumbar fascia or within hyaluronan-rich loose connective tissue between fascial layers [14,37]. Shear wave elastography, in turn, reflects local apparent tissue stiffness under the tested loading and imaging conditions, which may be determined more strongly by collagen architecture, layer tension, posture, probe-tissue interaction, and local mechanical state than by short-term systemic water availability alone [15,39]. Therefore, the evident reduction in urine specific gravity confirms a systemic urinary hydration response, but it does not demonstrate that fascial tissue hydration changed, nor that any local extracellular matrix change was large enough or rapid enough to modify shear-wave elastography-derived stiffness within the present sampling window.

The primary null finding for shear wave elastography is biologically plausible when interpreted against current fascial literature. Contemporary reviews of fascia emphasize that collagen architecture is a major determinant of load-bearing behavior, whereas hyaluronan-rich loose connective tissue is more directly involved in lubrication and interlayer gliding [8,39,40]. These concepts make hydration a plausible modifier of fascial function, but they do not establish that a short-lived systemic water load should promptly change in vivo thoracolumbar stiffness [8,39,40]. Human imaging studies have more consistently shown thoracolumbar fascial mechanical differences in relation to chronic low back pain, posture, movement, stretching, and myofascial intervention than in relation to acute systemic hydration [3,16,17,19,41]. That interpretation is strengthened by prior work showing that shear wave elastography can reproducibly quantify thoracolumbar or deep fascial stiffness and detect posture- or movement-related changes, which argues against a simple explanation based solely on measurement insensitivity [12,16,42]. However, the present data should not be interpreted as confirming, refuting, or substantially revising hydration-based models of fascial biomechanics as a whole. Rather, they show that a single acute 2 L oral water-loading protocol in healthy adults under resting conditions was not accompanied by detectable short-term change in thoracolumbar fascia stiffness measured by shear wave elastography. Whether local extracellular matrix hydration, hyaluronan organization, fascial gliding, or stiffness would respond under different baseline hydration states, longer exposure windows, symptomatic tissue conditions, or combined hydration-and-loading protocols remains unresolved [11,43].

The hydration-marker results clarify an important aspect of the intervention. The large decline in urine specific gravity confirms that the water-loading protocol changed hydration-related physiology over the expected time frame, which is consistent with prior work showing that urine specific gravity is a responsive hydration biomarker and can decrease within roughly 45–60 min after fluid ingestion in mildly hypohydrated adults [38,44,45]. By contrast, the absence of detectable change in intracellular water, extracellular water, and the extracellular-to-intracellular water ratio is compatible with the more heterogeneous literature on acute bioimpedance responses [22,46,47,48]. Some studies have reported little or no short-term change in impedance-based estimates after acute drinking, whereas others have observed shifts that depend on fluid composition, posture, measurement timing, and the specific prediction equations embedded in the device [22,46,47]. Because bioimpedance-derived fluid compartments are model-based rather than direct tissue measurements and remain sensitive to protocol conditions, the dissociation between urine specific gravity and bioimpedance in our data is unsurprising [46,47,48]. The most parsimonious interpretation is that urine concentration responded rapidly to the intervention, whereas whole-body compartment estimates did not shift enough, or not in a way that this protocol could detect, within the same short interval. Importantly, neither urine specific gravity nor bioimpedance directly verifies hydration of the thoracolumbar fascia itself. Consequently, the present study can establish that the oral water load produced a urinary hydration response, but it cannot determine whether local fascial water content, hyaluronan hydration, interlayer viscosity, or extracellular matrix fluid distribution changed during the observation period [10,46,47].

The fixed 2 L dose also requires interpretation as an absolute water-loading challenge rather than as an equivalent physiological dose for every participant. In the present sample, body mass and BMI varied meaningfully, so the same 2 L volume would have corresponded to different volumes per kilogram of body mass and probably to different fractions of total body water across individuals [30,31]. This heterogeneity may have increased inter-individual variability in urinary, bioimpedance, and stiffness responses and could have reduced sensitivity for detecting a dose-dependent fascial response, if such a response exists. Conversely, the marked group-level reduction in urine specific gravity indicates that the protocol was sufficient to perturb systemic urinary hydration at the sample level, but it should not be interpreted as evidence that all participants received an equivalent tissue-level hydration stimulus [24,38].

The exploratory association between the early change in urine specific gravity and the early change in mean thoracolumbar fascia stiffness should be interpreted cautiously. There is a biologically credible rationale for testing such an association, because hyaluronan-rich fascial layers contribute to lubrication and gliding and their physicochemical behavior is thought to depend on local water balance and molecular organization [8,39,40]. However, the lack of any omnibus temporal effect on shear wave elastography, the absence of point-specific changes, and the disappearance of the association by T2 indicate that this isolated early correlation is insufficient evidence for a stable coupling between systemic hydration and thoracolumbar fascial stiffness. One reasonable interpretation is that urine dilution and fascial mechanical behavior operate on different compartments and timescales, with systemic urinary biomarkers acting as imperfect surrogates for local extracellular matrix conditions [8,39,44]. An alternative explanation is that the early association reflects inter-individual variability around a very small signal rather than a reproducible physiological link, which is consistent with the trivial sample-level changes in stiffness observed across all three assessments.

This study has several limitations that should guide interpretation and future research. The principal limitation is the absence of a parallel non-hydration or placebo control condition. Although the within-participant repeated-measures design reduced between-person variability and all assessments were standardized, it cannot fully exclude time-related measurement effects, repeated-positioning or repeated-probe effects, regression to the mean, or uncontrolled physiological variation across the T0, T1, and T2 sampling window [25]. Accordingly, the present findings should be interpreted as showing no detectable within-participant change in thoracolumbar fascia stiffness after the tested hydration protocol, rather than as definitive evidence that hydration has no causal effect on fascial mechanical behavior. A parallel control group was not included because this initial study prioritized standardized within-participant physiological characterization after a supervised oral water load. However, future studies should use randomized crossover or controlled parallel-group designs with a non-hydration, sham-hydration, or time-matched comparator condition to isolate hydration-specific effects more rigorously. In addition, although the SWE protocol used standardized participant positioning, anatomical landmarks, ROI placement, and image-quality criteria, the study did not include a separate within-session or between-session repeatability substudy. Therefore, measurement precision in this sample cannot be quantified beyond the observed variability and previously published thoracolumbar fascia SWE reliability estimates [12].

A second limitation is that the intervention used a fixed absolute volume of 2 L rather than a dose normalized to body mass, baseline hydration status, sex, body composition, or estimated total body water. This approach was chosen to standardize the experimental challenge and ensure that all participants received the same externally administered water load, but it does not imply that the intervention represented an equivalent physiological stimulus for all individuals. Because total body water and body-water distribution vary with sex, adiposity, and body composition, the same absolute water volume may produce different relative fluid loads, renal responses, and compartmental distribution patterns across participants [30,31]. This may have contributed to inter-individual variability in urine specific gravity changes and may partly explain why a clear urinary response was not accompanied by detectable changes in bioimpedance-derived fluid compartments or thoracolumbar fascia stiffness. The present design therefore cannot evaluate dose–response effects, threshold effects, or nonlinear responses to smaller, larger, repeated, or individualized hydration strategies. Future studies should consider body-mass-normalized or total-body-water-normalized hydration protocols, dose-ranging designs, and stratification by baseline hydration status or body-composition phenotype.

Third, the study population was restricted to healthy adults without recent thoracolumbar or lumbar pain, limiting generalizability to clinical or physiologically distinct groups such as patients with chronic low back pain, older adults, athletes, individuals with chronic hypohydration, or persons with metabolic, inflammatory, medication-related, or connective-tissue conditions that could modify fluid regulation or fascial mechanical behavior [3,17]. Moreover, hydration was characterized using urine and bioimpedance markers rather than direct tissue-level measures, so the study cannot determine whether local fascial water content, hyaluronan hydration, or interlayer extracellular matrix fluid distribution changed despite stable stiffness. Future studies should therefore examine controlled hypohydration and rehydration paradigms, individualized or dose-ranging hydration protocols, symptomatic or high-load populations, direct or regionally targeted measures of fascial hydration and gliding, and protocols that test whether repeated hydration exposure interacts with movement or local fascial treatment over longer time scales.

From an applied perspective, the present findings should be restricted to the specific experimental context studied since healthy adults were assessed at rest after a single supervised 2 L oral water-loading protocol. Under these conditions, acute water ingestion was not accompanied by an immediate reduction in thoracolumbar fascia stiffness. These findings should not be extrapolated to patients with chronic low back pain, athletes exposed to high mechanical or thermal load, older adults, individuals with chronic low-intake dehydration, or populations with altered body composition, medication use, metabolic status, or connective-tissue pathology [3,17]. Such populations may differ in baseline fascial mobility, tissue stiffness, extracellular matrix composition, fluid regulation, and response to combined hydration and mechanical loading [6,8]. Therefore, the present results do not support recommending a single acute water load as an isolated strategy to reduce thoracolumbar fascia stiffness in healthy resting adults, but they do not address whether hydration status may interact with exercise, manual therapy, chronic tissue state, or rehabilitation exposure in clinically relevant populations.

## 5. Conclusions

In this prospective repeated-measures study of healthy adults, acute ingestion of 2 L of water produced a clear urinary hydration response but did not change thoracolumbar fascia stiffness, either globally or at any of the four standardized measurement points, and it did not materially alter bioimpedance-derived fluid distribution indices. Because no non-hydration or placebo control condition was included, the findings cannot exclude time-related measurement effects or other uncontrolled physiological variation and should not be interpreted as a definitive controlled estimate of the causal effect of hydration. Rather, the data indicate that, under standardized resting conditions, a 2 L oral water-loading challenge was not accompanied by detectable short-term within-participant modification of thoracolumbar fascial stiffness. Future randomized crossover or controlled parallel-group studies are required to determine whether hydration-specific effects emerge under different baseline hydration states, longer exposure windows, symptomatic conditions, or protocols combining hydration with local mechanical loading or movement.

## Figures and Tables

**Table 1:** Baseline characteristics of the analyzed sample.

Characteristic	Value
Age (years)	34.3 ± 7.6
Body mass (kg)	87.4 ± 14.8
Height (cm)	174.3 ± 9.2
Body mass index (kg/m^2^)	29.0 ± 5.9
Male sex, *n* (%)	28 (56.0)
Female sex, *n* (%)	22 (44.0)

Data are mean ± standard deviation unless otherwise indicated.

**Table 2:** Time course of hydration markers and mean thoracolumbar fascia stiffness.

Outcome	T0	T1	T2	F (df1, df2)	*p*	ηp^2^
Urine specific gravity	1.016 ± 0.002	1.006 ± 0.002	1.014 ± 0.002	290.175 (2, 98)	<0.001	0.856
Intracellular water (%)	30.99 ± 0.69	30.98 ± 0.70	31.00 ± 0.69	0.814 (2, 98)	0.446	0.016
Extracellular water (%)	18.01 ± 0.28	18.01 ± 0.31	18.00 ± 0.28	0.974 (2, 98)	0.381	0.019
Extracellular-to-intracellular water ratio	0.361 ± 0.001	0.361 ± 0.002	0.361 ± 0.002	0.103 (2, 98)	0.902	0.002
Mean thoracolumbar fascia stiffness (kPa)	45.88 ± 2.01	45.87 ± 2.01	45.89 ± 2.02	0.671 (2, 98)	0.514	0.013

Values are mean ± standard deviation. Omnibus tests are one-factor repeated-measures analyses of variance across T0, T1, and T2; df: degrees of freedom.

**Table 3:** Pairwise changes in urine specific gravity and mean thoracolumbar fascia stiffness.

Outcome	Contrast	Mean Change	95% CI	Cohen’s dz	Holm-Adjusted *p*
Urine specific gravity	T1–T0	−0.010	−0.012 to −0.010	3.394	<0.001
Urine specific gravity	T2–T0	−0.002	−0.004 to −0.001	0.673	<0.001
Urine specific gravity	T2–T1	0.008	0.007 to 0.009	2.719	<0.001
Mean thoracolumbar fascia stiffness (kPa)	T1–T0	−0.020	−0.060 to 0.020	0.128	>0.999
Mean thoracolumbar fascia stiffness (kPa)	T2–T0	0.000	−0.030 to 0.040	0.035	>0.999
Mean thoracolumbar fascia stiffness (kPa)	T2–T1	0.020	−0.030 to 0.080	0.130	>0.999

Mean changes are expressed as the later minus the earlier time point. Negative values therefore indicate decreases over time, and positive values indicate increases.

**Table 4:** Thoracolumbar fascia stiffness at the four measurement points.

Point	T0, kPa	T1, kPa	T2, kPa	F (df1, df2)	Holm-Adjusted *p*	ηp^2^
P1	45.98 ± 2.08	45.95 ± 2.09	45.98 ± 2.14	0.313 (2, 98)	>0.999	0.006
P2	45.72 ± 2.10	45.72 ± 2.12	45.73 ± 2.13	0.012 (2, 98)	>0.999	0.000
P3	45.99 ± 2.16	45.99 ± 2.18	46.00 ± 2.19	0.038 (2, 98)	>0.999	0.001
P4	45.85 ± 1.96	45.80 ± 1.94	45.86 ± 1.95	0.767 (2, 98)	>0.999	0.015

Values are mean ± standard deviation. The primary two-factor repeated-measures model for shear wave elastography yielded F(2,98) = 0.671, *p* = 0.514, ηp^2^ = 0.013 for time; F(3,147) = 2.147, *p* = 0.097, ηp^2^ = 0.042 for measurement point; and F(6,294) = 0.195, *p* = 0.978, ηp^2^ = 0.004 for the time-by-point interaction.

## Data Availability

Data is available upon reasonable request to the corresponding author.

## Abbreviations

The following abbreviations are used in this manuscript:

ANOVA	Analysis of Variance
SWE	Shear wave elastography measurement of thoracolumbar fascia stiffness
